# Experiences With Online Teaching and Psychological Adjustment of High-School Students at the Onset of the COVID-19 Pandemic in Croatia

**DOI:** 10.3389/fpsyg.2021.647991

**Published:** 2021-07-22

**Authors:** Vesna Buško, Petar Bezinović

**Affiliations:** ^1^Department of Psychology, Faculty of Humanities and Social Sciences, University of Zagreb, Zagreb, Croatia; ^2^Independent Educational Consultant, Rijeka, Croatia

**Keywords:** COVID-19, educational stressors, emotional health, adaptation, Croatian high-school students, gender and grade differences

## Abstract

Vastly changed schooling arrangements due to the COVID-19 crisis have generally limited the opportunities and resources for schools to provide necessary psychological and other support to their students. Given this, all parties involved in the schooling system need to understand the kinds of experiences students have via distance learning and how students adapt to novel living and studying conditions. This study examines the relevant sources of stress that students encounter with regard to online classes, and the frustrations they face due to living in social isolation, as well as how these stressors relate to the measures of students’ emotional wellbeing and psychological adjustment. Data were collected in April 2020 via an online questionnaire administered to a sample of 4,492 students (62.6% female) attending all four grades of high school within two Croatian counties. The results point to the differential effects of particular aspects of online teaching practices on the stressful experiences of students. Furthermore, the results of multivariate analysis of variance and canonical discriminant analyses demonstrated notable gender and grade differences in the structure of students’ emotional and behavioral adaptation to the health crisis. Female students and those predominantly studying at the highest grade reported higher frequency and intensity of short-term emotional and psychosomatic difficulties. Male students in the final grade year scored highest on the dimension defined by the number and intensity of online teaching stressors. The results are interesting in light of hypothetical accounts of adjustment mechanisms based on existing stress theories. Moreover, the findings serve as a basis for teachers’ self-reflection and self-evaluation of schools, which can then lead to designing specific programs of support in learning and adaptation to the new living and studying conditions.

## Introduction

The COVID-19 pandemic is creating significant turbulence in the lives of individuals and societies worldwide. This health emergency has critically impacted all segments of contemporary societies, including every level of the schooling system. The vital implementation of various social distancing measures has meant the partial or total closure of schools and a shift from regular face-to-face teaching to online teaching. This major alteration in instruction and learning practices has impacted the vast majority of educational institutions worldwide (Alemany-Arrebola et al., 2020; Rettie and Daniels, 2020; Toquero, 2020; UNESCO, 2020; Yan, 2020).

From the perspective of youth mental health care, the importance of schools cannot be overemphasized. Schools play a unique role in ensuring a healthy and safe environment for their students. In this sense, schools provide necessary support for the development of individual capacities and other personal qualities of students. Realization of potential is a basis for the productive and healthy life of any young person, and this is frequently stressed as a priority of modern education (Wentzel, 1998; Anderman, 2002; Klem and Connell, 2004; Shochet et al., 2006; Bezinović and Tkalčić, 2017). To achieve this goal, schools are expected to foster students’ cognitive, socio-emotional, cultural, and physical growth in accordance with the capabilities and interests of each individual student. Significant changes to schooling arrangements due to the COVID-19 crisis have generally limited schools’ opportunities and resources to provide psychological and other support to their students. In such circumstances, all parties involved in the schooling system need to understand students’ experiences with distance learning and how they adapt to novel living and studying conditions.

Considerable empirical evidence has already been collected on the diverse emotional, health, and other consequences of the global COVID-19 crisis (e.g., Cachón-Zagalaz et al., 2020; Rettie and Daniels, 2020; Saha et al., 2020; Taylor et al., 2020; Valadez et al., 2020; Xin et al., 2020). Studies have reported heightened levels of stress, and unfavorable psychological states such as negative affect, symptoms of anxiety and depression, and violent, hostile, and addictive behavior, all of which have been found in the general population as well as among students and adolescents since the onset of the global pandemic (Alemany-Arrebola et al., 2020; Dubey et al., 2020; Li H. Y. et al., 2020; Li X. et al., 2020; McCracken et al., 2020; Richter, 2020). These findings are not surprising given the huge cumulative scientific evidence about general stress, health, and adaptation processes (see, e.g., Lazarus, 2000; Schneiderman et al., 2005). Moreover, while the COVID-19 health risk can be a severe stressor by itself, all the rapid and significant changes in the daily lives of people arising from the emergency have presented additional and potentially serious sources of stress.

Adolescents and high-school students seem to have been especially affected by these abrupt changes to daily life, predominantly due to school closures. On the one hand, the usual structure of school activities and daily routines has been lost; on the other hand, the multitude of experiences that normally occur through intensive social interactions with peers and authorities outside of family have become scarce. Due to features specific to their developmental stage, late adolescents are probably the most sensitive to these various kinds of social deprivation (Wentzel, 1998; Anderman, 2002; Klem and Connell, 2004; Roviš and Bezinović, 2011; Wentzel et al., 2018; Kuhfeld et al., 2020). In addition, such threats might be even more intensely experienced by students in the final grade year of high school. This appears to be primarily due to the practical circumstances of living and studying during the so-called first COVID-19 wave, which led to uncertainties about completion of schooling, the timing and form of final graduation exams, and the prospects of university admission for all individuals planning to continue into higher education.

Therefore, it is interesting and of practical importance to focus on high-school students’ stressful experiences in order to gain insights into how they are coping with profoundly changed living and learning circumstances. This study was begun in the initial weeks of online schooling in Croatia, and it is the first piece of research to provide systematic empirical data from Croatia on the effects of school closure as a source of stress and the assessment of online teaching from the students’ perspective. Consequently, our findings served as a platform for creating specific education policy measures by the Croatian Ministry of Science and Education.

We conceptualized the study based on the well-known Lazarus transactional stress and coping perspective (Lazarus and Folkman, 1984; Lazarus, 2000), drawing particularly on its cognitivist and contextual understanding of the stress processes. The cognitivist perspective of the Lazarus theory holds that not only objective circumstances, but also subjective appraisals of situations, are the key determinants of how an individual handles situational demands. Hence, adequate predictions of adjustment to specific stressful circumstances require taking into account the unique adaptive demands of the situation and the associated cognitive appraisals used by individuals in interpreting the personal meaning of these demands. Moreover, the contextual character of the theory is manifested in the notion that transient situation-based factors, rather than personal dispositions, are assumed to shape individuals’ responses, choice of coping with situational demands, and resulting adaptation outcomes. Relatively stable personal, social, and/or environmental influences are expected to play only an indirect role in this process and are regarded as the antecedents of situational appraisals and coping responses.

Relying on this general stress and coping framework, our study focused on the issues of stressfulness or, in terms of Lazarus’s theory, the primary cognitive appraisals of various aspects of studying and living at the onset of the pandemic, and the relationships of these appraisals with indices of wellbeing in high-school students. We aimed to explore the relevant sources of stress that students encounter with regard to online classes and the frustrations of living in social isolation, as well as how these stressors relate to the measures of students’ emotional wellbeing and psychological adjustment. As the main study question, we intended to examine the structure of differences in reported stressful experiences and the indices of emotional and behavioral adaptation to the changed living conditions as a function of the grade level and gender of students.

Based on plentiful theoretical and empirical literature in the area of stress and coping (e.g., Endler et al., 2000; Lazarus, 2000; Somerfield and McCrae, 2000; DeLongis and Holtzman, 2005; Scherer and Moors, 2019), we hypothesized substantial positive relationships between reported sources of stress and indices of emotional distress, as well as negative relations of these variables with the measure of effective adjustment to stressful situations. Furthermore, we expected significant gender and grade-level differences in the extent and intensity of stress experiences generated by sudden changes in living and learning conditions. No expectations were stated with regard to specific sorts of events being experienced by students as more or less stressful, although generally higher levels of stress appraisals and more frequently occurring indices of emotional distress were predicted to be reported more by female students than by male students.

## Methods

This study was designed as a response to the initiative of the professional organizations of school psychologists of the Istrian County of the Republic of Croatia, following recommendations by the Ministry of Science and Education of the Republic of Croatia. The principal objective of this initiative was to gain an insight into the experiences of high-school students with the immensely changed living and learning circumstances due to the onset of the COVID-19 pandemic. The administration of the study was accomplished as part of regularly scheduled school activities. A multivariate cross-sectional correlational design was implemented to examine the main study questions.

### Participants

A total of 4,492 students (62.6% female) attending 21 high schools in the regions of Istria and Primorsko-Goranska participated in the study. The sample consisted of students from the first (28.9%), second (26.1%), third (25.2%), and fourth (19.8%) grades in secondary schools, covering the age range of 15–19 years. Students attending grammar schools and professional high schools from these regions took part in the study. Given the sample size and general demographic figures, the sample is a good representation of the high-school student population in the two regions and, to a considerable extent, the Croatian adolescent population of that age. The sample is not very well balanced by gender, as it consists of a somewhat larger proportion of females than would be expected by population values.

### Instruments and Procedures

The data were collected during April 2020 via an online questionnaire prepared using the SurveyGizmo (now Alchemer) Internet platform. The entire instrument was designed and adapted specifically for the present study, with selected scales and items being taken in more or less equivalent (original) form from the extensive questionnaire entitled “Kako si? [How are you?]” which was developed and extensively validated within the long-term project on “Risk behaviors, lifestyles, and adjustment of highs-school students” (2001–2019) led by the second author of this study (e.g., Bezinović and Buško, 2008; Bezinović and Malatestinić, 2009; Roviš and Bezinović, 2011; Bezinović, 2016; Bezinović and Tkalčić, 2017). This applies to most of the questionnaire content not explicitly related to the events or stressors due to the current pandemic. The questionnaire consisted of five main sections:

1. *Stressful experiences with distance learning*. The first part of the questionnaire comprised 14 items describing possible sources of stress related to different aspects of online schooling. Students were to appraise how stressful they personally found each of the presented problems. Answers were given with reference to the preceding 2-week period using a Likert-type scale ranging from “not at all stressful” (0) to “extremely stressful” (3). The internal consistency of this group of 14 items as measured by Cronbach’s alpha was 0.89.
2. *Hindrances of life in social isolation*. These five items represented possible obstacles and worries students might have faced due to social isolation during the prior 2 weeks. The same four-point Likert-type response format was used. Cronbach’s alpha for this set of questions was 0.78. The content of all the items covered by the first two sections of the questionnaire (i.e., this one and that described above) is listed in Table 1.

**TABLE 1:** Types and intensity of stressful experiences with distance learning and life in social isolation by gender.

Stressful experiences with distance learning	Female	Male
Demanding school assignments in various subjects	69,7%	59,3%
Acquiring difficult study contents	56,4%	43,9%
Meeting deadlines to complete all study tasks	52,6%	46,1%
Uncertainty about return to schools	52,0%	35,8%
Missing live lectures	51,6%	39,0%
Handling many “posts” in virtual classrooms	44,2%	35,1%
Learning uninteresting content	42,9%	38,2%
Learning unaided from many different sources	40,5%	31,7%
Missing concentration during online lectures	35,9%	27,0%
Acquiring digital technology needed for online learning	27,9%	20,1%
Online communication with teachers	20,8%	17,3%
Inadequate conditions for learning (e.g., missing quiet room, free PC, internet access)	18,9%	15,3%
High parental expectations regarding school obligations	16,3%	17,2%
Receiving feedback from teachers	15,7%	14,1%

**Hindrances of life in social isolation**		

Restrictions in meeting friends live	81.4%	72.6%
Missing free-time activities	65.0%	59.7%
Confinement at home	61.4%	46.9%
Concern that I will not achieve the kind of school success I want	58.1%	41.4%
Spending all day in the same space as family members	38.5%	32,0%

**N* varies to some extent depending on the total number of participants with an answer on each item. Percentages denote % of subsamples with answers 2 (‘considerably’) and 3 (‘extremely stressful’) to each item. Mean differences/differences in proportions for all but the two last items are significant with *p* < 0.01. Girls demonstrate higher level of stress experiences.*

3. *Threats to emotional health*. Ten items were selected to cover a range of unpleasant emotional states, including anxiety, sadness, loneliness, and psychosomatic symptoms (e.g., “I felt helpless”). Students appraised, using a four-point frequency scale from “never” (0) to “very often” (3), how often they had experienced these states in a described manner during the previous 2 weeks. Cronbach’s alpha for this set of questions was 0.88.
4. *Adjustment to new life circumstances*. Six items examined general adaptability in an emergency situation, including feelings of competence, optimism, and contentment (e.g., “I was able to focus on learning and my homework”). This measure was used as a composite indicator of emotional and cognitive wellbeing following its recently proposed conceptual definition (Ross et al., 2020). Appraisals of items in this section were done using a four-point Likert-type format ranging from “does not apply to me at all” (0) to “fully applies to me” (3), with reference to the preceding 2-week period. Cronbach’s alpha for these items was 0.78.
5. Several pieces of basic sociodemographic data were also collected: information on the region (Istria or Primorsko-Goranska), the mother language of participants (Croatian or Italian), type of school, school level (first to fourth grade of secondary school), and gender.

The schools received the link through which each student could access the e-questionnaire and take part in the study. In schools where teaching was in Italian, appropriate translations of the questionnaires were administered. The study was conducted with the approval of liable county bodies and in accordance with the regulations of the Croatian Ethical Codex of Research on Youth, and the Research Ethics Codex of the Department of Psychology, University of Zagreb. Participation in the study was anonymous and fully voluntary with an option to resign at any time during completion of the questionnaire. By accepting to fill in the instrument, students gave their consent to take part in this study. Apart from the time it took to answer several open-ended questions, which we do not analyze in detail in this study, the completion of the questionnaire took approximately 15 min.

### Data Analysis

Along with descriptive and reliability statistics of all the study variables, we performed item-level principal component analyses with promax rotation as an auxiliary check of the structure of the data to be of use in defining composite study variables. Multivariate analysis of variance (MANOVA) and canonical discriminant analyses were conducted to answer the main study questions. All the analyses were done using IBM SPSS statistical software, v. 24.0.

## Results

The main study results are based on composite scale scores on the key variables analyzed in response to the study questions. An account of these findings is preceded by a brief outline of students’ ratings of a range of specific sources of stress they might have experienced within a 2-week period. Table 1 (with figures given separately by gender) presents elementary descriptive data on sets of items describing stressors potentially generated by conditions of online learning and life in social isolation.

Following the proportions of students who rated different distance-schooling situations as considerably and/or extremely stressful, the most salient source of stress, in both female (69.7%) and male (59.3%) participants, appears to have been students’ overload due to many assignments in various subjects. Likewise, acquiring difficult study contents (56.4% of female students; 43.9% of male students) and meeting deadlines for completion of all study tasks (52.6% of female students; 46.1% of male students) were also reported as rather stressful. Other aspects of distance learning were reported to be somewhat less stressful, with items describing parental expectations and getting feedback from teachers being rated as the least taxing by both genders (14.1 to 17.2%). As can be seen, female students generally reported higher levels of stress experiences than did male students, except for the two situations appraised as least stressful where no significant gender differences were observed.

All five presented aspects of life in social isolation were rated as considerably or extremely stressful by a substantial number of students, with obvious differences observed between genders and specific sources of frustration. Again, girls showed significantly higher levels of stressfulness on all items compared to boys. Based on the proportions obtained, restrictions in meeting friends and hanging out appears to have posed the largest difficulties to both female (81.4%) and male (72.6%) students. Conversely, conditions of spending most of the time with family members in the same area was rated as the least demanding aspect by both genders (38.5% of female students; 32% of male students).

The main descriptive statistics of the four composite study variables are shown in Table 2. Comparing the mean composite scores on the two scales comprising different sources of recent situational pressures, limitations of life in social isolation seem to elicit on average visibly higher level of stress experiences among students (*M* = 1.71, *SD* = 0.76) than the various conditions of distance learning (*M* = 1.19, *SD* = 0.61). The results on the emotion scale, used as a measure of threats to emotional health, suggest that the level or rate of recurrence of short-term unpleasant emotional states in students was on average relatively low (*M* = 1.07, *SD* = 0.68), with the mean score corresponding to the item scale point of “rarely.” Moreover, the results obtained on the remaining measure of overall adaptability suggest that high-school students are on average reasonably well adjusted to these sudden and unfamiliar living and studying circumstances (*M* = 1.86, *SD* = 0.62).

**TABLE 2:** Main descriptive statistics and intercorrelations of the composite scale scores.

Scales	*N*	Mean	*SD*	Skewness	Kurtosis	Correlation			*k*	Cronbach alpha
						2.	3.	4.		
(1) Distance learning	4483	1.19	0.61	0,389	–0,244	0,582	0,507	–0,481	14	0.89
(2) Emotional distress	4449	1.07	0.68	0,552	–0,293	–	0,542	–0,454	10	0.88
(3) Social isolation	4418	1.71	0.76	–0,269	–0,730		–	–0,296	5	0.78
(4) Adjustment to situation	4426	1.86	0.62	–0,533	0,289			–	6	0.78

**N* varies to some extent depending on the total number of participants with complete answers on all items of the respective scales. Summative scores on all scales are divided by the number of items to simplify scale comparisons and interpretations; total scores on all scales vary in the observed range of 0-3; *Scales*: (1) Stressful experiences with distance learning, (2) Threats to emotional health: emotional and psychosomatic problems, (3) Hindrances of life in social isolation, (4) Adjustment to new life circumstances; *k*, number of items per scale; all correlations are significant at *p* < 0.001.*

Both measures of stressful experiences showed substantially high positive correlations with scores on the scale for short-term emotional state, and somewhat lower, albeit also significant, negative correlations of moderate size with the measure for overall adjustment to the situation. The shape of distributions of variables and the indices of asymmetry and kurtosis indicates a certain departure from normality; however, these values varied within the tolerable range and, given the rather large sample and subsample sizes, the implementation of further multivariate data analyses appeared to be warranted.

To examine the differences in reported stressful experiences as well as in emotional and behavioral reactions to novel living conditions as a function of gender and the grade level of students, we performed MANOVA, implementing the factorial 2 × 4 design (e.g., Huberty and Olejnik, 2006). The analysis revealed significant main effects of both gender (Wilks’ Λ = 0.85, *F*[4, 4271] = 187.92, *p* < 0.001, partial η^2^ = 0.150) and grade (Wilks’ Λ = 0.96, *F*[12, 11300.29] = 11.630, *p* < 0.001, partial η^2^ = 0.011, Roy’s GCR = 0.032, *F*[4, 4273] = 34.51, *p* < 0.001, partial η^2^ = 0.031), with the gender x grade interaction found to be marginally significant (Wilks’ Λ = 0.99, *F*[12, 11300.29] = 1.57, *p* = 0.092, Roy’s GCR = 0.003, *F*(4, 4273) = 3.199, *p* = 0.012, partial η^2^ = 0.003).

Following the outcomes of the multivariate tests, canonical discriminant analysis was performed as a standard procedure accompanying MANOVA to explain the nature of multivariate group differences (e.g., Bray and Maxwell, 1985; Nesselroade and Cattell, 1988). Both factors of gender and grade level were taken into account to define the variable of group membership in discriminant analysis, thereby enabling a thorough account of observed variability in stress appraisals and reactions to stressful circumstances. The same four discriminant variables were used (two composite measures of appraisals of stressors, that is, distance learning and social isolation, and outcome measures of emotional distress and adjustment to situation) and groups were defined by gender and cohorts of students (hence, eight groups were created in total). The analysis yielded two significant canonical discriminant functions accounting for almost all examined variability between the eight groups of students (99.4% of intergroup variance) in the space of the four stress and adjustment discriminant variables (Table 3).

**TABLE 3:** Significance tests, eigenvalues (λ), Wilks’ Λ, and canonical correlations of the discriminant functions for groups defined by gender and cohorts of students.

Function	Canonical correlation	Eigenvalue (λ)	Wilks’ Λ	Chi-Square	*df*	*p*
1	0.411	0.203	0.815	874.89	28	<0.0001
2	0.135	0.019	0.980	84.55	18	<0.0001
3	0.032	0.001	0.999	6.09	10	ns

**df*, degrees of freedom; *p*, significance of the Chi-Square test.*

All the parameters presented in Table 3 indicate significant though not particularly marked differences between the eight groups of students, with the most discriminant power being attributable to the first discriminant function (91.1% of total intergroup variability). As shown in the table, canonical correlation and Wilks’ lambda obtained even for the first discriminant function point to moderate intergroup differences (*r* = 0.411, Λ = 0.815). The meaning of the derived functions and relative positions of the eight groups in the two-dimensional space are presented in Tables 4, 5.

**TABLE 4:** Standardized discriminant function coefficients and structure coefficients for the two significant canonical discriminant functions with groups defined by gender and cohorts of students.

	Function 1		Function 2	
Discriminant variable	Standardized coefficients	Correlations	Standardized coefficients	Correlations
(1) Distance learning	–0.046	0.402	1.293	0.854
(2) Emotional distress	1.164	0.925	–0.420	0.114
(3) Social isolation	–0.035	0.422	–0.279	0.103
(4) Adjustment to situation	0.412	–0.107	0.127	–0.218

*Structure coefficients are computed as pooled within-groups correlations between discriminant variables and standardized canonical discriminant functions; *Variables*: (1) Stressful experiences with distance learning, (2) Threats to emotional health: emotional and psychosomatic problems, (3) Hindrances of life in social isolation, (4) Adjustment to new life circumstances.*

**TABLE 5:** Positions of groups centroids on the two significant canonical discriminant functions.

			Function 1	Function 2
Group			Group centroid	Group centroid
n	Gender	Grade		
(1) 764	F	1	0,153	–0,138
(2) 696	F	2	0,369	–0,024
(3) 676	F	3	0,368	–0,046
(4) 564	F	4	0,474	0,138
(5) 476	M	1	–0,739	–0,158
(6) 415	M	2	–0,558	–0,016
(7) 407	M	3	–0,553	0,162
(8) 284	M	4	–0,287	0,322

**N* = 4282 (cases with missing gender and/or grade codes and those with missing value on at least one discriminant variable are excluded from the analysis); *n*, group sizes; F, female; M, male.*

The first discriminant function is primarily defined by scores on the emotional distress measure, whereas the contribution of the remaining scales to the discriminant scores on this function is sizably smaller or negligible. High scores on this function are attained by those who reported frequent occurrence of unpleasant emotional states or various psychosomatic symptoms within the 2-week period. The second discriminant function is predominantly defined by the measure of stressful experiences with distance learning; here too, the role of the other discriminant variables is negligible. Participants who reported high levels of stress experienced due to various aspects of online schooling attained high scores on the second discriminant function.

Analogous to the discriminant function parameters, the obtained range of group centroid values, presented in Table 5, demonstrates that the groups of students differ to a considerably larger extent on the first discriminant function than they do on the second. The first function opposes male and female groups of students, with the group of male students attending the first grade of high school scoring lowest (C_5_ = -0.739), and the female group attending the final grade of high school having the highest centroid score on the function (C_4_ = 0.474). Centroids of most of the groups on the second function are positioned around zero, with the only group standing out with a somewhat higher centroid score is that comprising the senior male students (C_8_ = 0.322).

## Discussion and Conclusion

This study was designed to examine the types and intensity of stress experienced and reported by high-school students in the course of distance learning and living in social isolation. In addition, the aim of the study was to investigate the relationships of stressor appraisals with the emotional wellbeing and psychological adjustment of students. We also examined the structure of grade and gender differences in students’ stress experiences and the specified emotional and behavioral outcome measures. The study hypotheses and interpretations are based on general stress and coping theory (Lazarus and Folkman, 1984; Lazarus, 2000) and the accumulated scientific evidence in the area of stress and adaptation mechanisms.

Descriptive analyses performed on the individual items reveal that a considerable proportion of students of both genders reported rather intensive stress experienced due both to different aspects of online learning and to frustrations of life in social isolation. Nevertheless, ample variations were observed among different sorts of stressors in terms of appraised intensity. Thus, the stressfulness of specific distance-schooling situations ranged from the least stressful source reported by around 15% of the sample for receiving feedback from teachers, to stressors reported by 50–70% of the sample, which include situations of uncertainty about return to schools, missing live lectures, acquiring difficult study contents, assignment overload, meeting deadlines, and so on. Further, the stressfulness of conditions of social isolation varied within a somewhat narrower range, from spending most of the time with family members, which was estimated as the least challenging by both female (38.5%) and male (32%) subsamples, to restrictions on meeting friends live, which was declared as a serious source of problem by the largest proportions of female (81.4%) and male (72.6%) students.

It should, however, be noted that the composite scale scores offer a somewhat different picture of the obtained results. Thus, based on the summative scores, both the amount of stress experienced due to situations of distance learning and the emotional distress experienced in the same period, used as a short-term outcome measure, seem to be rather modest. The same applies to the remaining outcome measure – overall adjustment to the current situation, which was used as a composite indicator of emotional and cognitive wellbeing. Students seem to have been reasonably well adjusted to the changes in life circumstances, including being mainly optimistic about the situation and generally capable of overcoming the difficulties and controlling their own behavior. Moreover, this study’s results on both outcome measures do not indicate a higher frequency or intensity of unpleasant emotions or other adjustment difficulties in students, compared to overall scores on analogous indices of wellbeing pertaining to practically the same high-school student population and obtained in prior and relatively recent studies that were conducted before the onset of the COVID-19 crisis worldwide (e.g., Bezinović and Tkalčić, 2017). While these are certainly positive and encouraging findings, which have also been reported in other studies (Zacher and Rudolph, 2020), adequate understanding of such a result requires taking into account the timing of the data collection. Our study was conducted at the very beginning of the COVID-19 crisis in Croatia, and most of the sample data were completed in early April 2020, which mainly corresponds to the timing of the second and third measurement points presented in Zacher and Rudolph’s (2020) longitudinal study. These authors found no changes in life satisfaction, positive affect, and negative affect in a sample from the adult German population between December 2019 and March 2020. However, a significant decrease in all three subjective wellbeing measures was detected in the same study for the data between March and May 2020. Similar inferences can be derived from other recent studies showing an increase in mental health difficulties during the pandemic, compared to previous general population data (Rettie and Daniels, 2020), increases in negative affect and symptoms of anxiety and depression across the confinement period among the Chinese college population (Li H. Y. et al., 2020), or increases in mental health symptomatology concerns recorded on social media in the 2-month COVID-19 period compared to a similar period in the previous year (Saha et al., 2020).

It should also be mentioned that the observed scores on all the variables in our study cover the full range of scale results, which, together with the other descriptive statistics, indicates large inter-individual variability in the examined stress-related measures. Both sets of results are interesting in terms of weighing the extent of pandemic-related psychological effects in specific populations (e.g., Alemany-Arrebola et al., 2020; Cachón-Zagalaz et al., 2020; Dubey et al., 2020; Li H. Y. et al., 2020; Li X. et al., 2020; Richter, 2020; Saha et al., 2020). Nevertheless, the individual differences are expected and understandable from the perspective of Lazarus’s transactional theory, especially considering its cognitivist character, which is manifested in the emphasis put on the evaluative cognitive processes in explaining stress and adjustment phenomena (Lazarus and Folkman, 1984). Hence, although all students are exposed to objectively comparable learning conditions, individual perceptions make the same situations more or less taxing, which consequently can lead to corresponding individual differences in adaptational outcomes.

The correlations between stress appraisal and outcome measures substantiate such interpretations based on Lazarus’s theory, and they support our hypotheses on the pattern of relationships among the main study variables. In other words, the theory foresees that the more the actual events or circumstances are appraised by a person as taxing and stressful, the higher the demands on his/her adaptive capacities are and, consequently, the lower the prospects for the adjustment to be effective are (see e.g., Lazarus and Folkman, 1984). Our results confirm the expected relatively high positive links between reported stress intensity, including measures of both categories of sources of stress, and the intensity of negative emotions as a short-term adaptational outcome measure. Likewise, the results confirm the negative, although somewhat lower, correlations of the stress intensity variables with the measure of effective adjustment to stressful situations. The observed differences in the size of correlations might be the result of the conceptual meaning of the outcome measures used; namely, both operationalizations were intended to serve as short-term outcome measures, at least according to the time frame given for answering the scales and specified in the instructions to the questionnaire. However, the measure of adjustment to situation includes items referring to, for example, sense of competence, self-efficacy, or optimistic views – that is, variables reflecting to some extent trait-like characteristics – so it may be less liable to transitory and contextual changes than is the emotional states measure.

Apart from being theoretically meaningful, our results are in line with the rich empirical literature on the general stress and adjustment processes (e.g., Endler et al., 2000; Lazarus, 2000; DeLongis and Holtzman, 2005; Schneiderman et al., 2005; Wentzel et al., 2018). Moreover, several other recent studies dealing with COVID-19 related stressors have reported relationships of equal sign and comparable size between a range of operationalizations of stress experiences and specific psychological states measures or variously defined behavioral, health, or performance outcomes (e.g., Alemany-Arrebola et al., 2020; McCracken et al., 2020; Valadez et al., 2020; Xin et al., 2020).

Lastly, the results demonstrate significant grade and gender differences in the analyzed set of stress and adjustment variables, with the observed multivariate differences attributable to gender being of a noticeably larger extent than those attributable to students’ grade. As shown by the results of the discriminant analysis, two derived latent dimensions accounted for the observed intergroup variability, with the first discriminant dimensions being defined principally by the scores on the emotional distress outcome measure, and the second function by the scores on the measure of stressors related to distance learning. As hypothesized, female students and those predominantly studying at the highest grade levels reported higher frequency and intensity of short-term emotional and psychosomatic difficulties. The results for the first function are expected and consistent with an extensive body of empirical literature showing the same direction of gender differences in a number of stress-related outcomes (Schneiderman et al., 2005; Alemany-Arrebola et al., 2020; Valadez et al., 2020). However, male students at the final grade level scored highest on the dimension defined by the intensity of online teaching stressors. These last findings, although not anticipated, are explicable due to senior students, at the time of this study, being exposed to several additional sources of potential stressors that were partly generated by uncertainties about the timing and form of final graduation exams, and partly by procedures related to university admissions during the pandemic for all individuals planning to proceed to higher education.

### Study Limitations and Suggested Research Lines

Several limitations of the present study should be mentioned. While the data collected in this study offer, in our opinion, theoretically and practically valuable information on important stress appraisal and short-term outcome-related variables, a thorough understanding of the stress processes elicited by conditions of studying during the pandemic would require taking into consideration other psychological attributes proven to be relevant factors in individuals’ responses and adaptation to stressful circumstances. These would include, for instance, certain personality dispositions, measures of cognitive efficacy, additional background and socio-environmental factors, and situation-specific coping mechanisms, along with measures of their effectiveness – that is, measures of the impact of these processes on short- and long-term educational, health, or other adaptation outcomes. Such a study would preferably seek longitudinal data as part of an adequate research design for testing hypotheses relating to stress and coping processes. Given the complex nature of the enduring COVID-19 pandemic, analyzing a role of other types of cognitive appraisals, and measures of perceived stressor controllability in particular, would be advantageous in clarifying the dynamics of school-related stress experiences and indices of wellbeing relationships during the course of the pandemic.

### Practical Implications

The insights into students’ experiences revealed in this study appear to be more or less directly applicable to educational practice; hence, they are probably useful in a broader context than that of the Croatian high-school system. First, different aspects of online schooling experiences appear to vary extensively in terms of reported stressfulness, suggesting that some features of the new teaching practices deserve more attention (e.g., the issue of the complexity and volume of weekly assignments that students are able to accomplish given the circumstances) than others (e.g., technical or communicational aspects) when planning alternative forms of teaching or upgrading existing ones.

Second, there are apparently large individual differences both with regard to the aspects of living or learning circumstances experienced by students as stressful, and in terms of the reported intensity of emotional and adjustment difficulties. These findings suggest that attempts to implement interventions aimed at alleviating the adverse effects of the current crisis would only make sense if they are highly individualized and adjusted to the specific concerns of particular students.

Third, the fact that the highest levels of stress were reported by senior students suggests that situations of uncertainty due to lack of information provided by relevant policy makers on important issues, such as how the state graduation exams will be implemented in the new circumstances, are probably more serious stressors than all the actual changes in living and learning conditions. As briefly noted earlier in this paper, this study was designed following an initiative by the school authorities and the regional organizations of school psychologists, so the objectives of such an endeavor identified and declared by these bodies were clearly of a practical nature. Upon completion of the study, each school received an extensive report with their own results along with detailed overall study results pertaining to the total sample. Thus, the study results served as a basis for self-reflection among teaching staff and self-evaluation by schools participating in the study. All the insights and comparisons with other schools provided opportunities for the schools for more informed decision-making on necessary adjustments required given the global change in health circumstances.

## Data Availability Statement

The raw data supporting the conclusions of this article will be made available by the authors, without undue reservation.

## Ethics Statement

Ethical review and approval was not required for the study on human participants in accordance with the local legislation and institutional requirements. Written informed consent from the participants’ legal guardian/next of kin was not required to participate in this study in accordance with the national legislation and the institutional requirements.

## Author Contributions

VB contributed to the conceptual design, literature research, data analysis, and writing of the manuscript. PB contributed to the conceptual design, data acquisition, data preprocessing and interpretation of results, and writing of the manuscript. Both authors contributed to the article and approved the submitted version.

## Conflict of Interest

The authors declare that the research was conducted in the absence of any commercial or financial relationships that could be construed as a potential conflict of interest.
